# Impact Assessment of Spatial Mismatch Between Grain Production and Consumption on Non-Point Source Pollution and Carbon Emissions from Grain Production in China

**DOI:** 10.3390/foods15101659

**Published:** 2026-05-09

**Authors:** Hui Yang, Stefan Sieber

**Affiliations:** 1 College of Public Administration, Nanjing Agricultural University, Nanjing 210095, China; 2 Leibniz Centre for Agricultural Landscape Research, 15374 Müncheberg, Germany; 3 Department of Agricultural Economics, Humboldt University of Berlin, 10117 Berlin, Germany

**Keywords:** spatial mismatch, grain production and consumption, non-point source pollution, carbon emissions, China

## Abstract

The spatial mismatch between grain production and consumption (SMGPC) significantly affects the non-point source pollution and carbon emissions from grain production (NPCE). Based on provincial panel data in China from 2000 to 2023, this study explored the direct impact and transmission mechanism of the SMGPC on the NPCE using fixed-effect and mediating-effect models. The results showed that China’s SMGPC intensified significantly during 2000–2023. The average SMGPC degree increased with fluctuations, with positive mismatch strengthening in the northern region and negative mismatch deepening in the southern region. All four NPCE indicators—non-point source pollution per unit sown area (NPA), carbon emissions per unit sown area (CEA), non-point source pollution per unit yield (NPY), and carbon emissions per unit yield (CEY)—peaked in 2016 and declined thereafter. The NPA and CEA showed net increases, whereas the NPY and CEY exhibited net decreases, presenting a similar spatial pattern of “high in the eastern region, low in the central and western regions”. The SMGPC degree drove NPCE indicators with obvious heterogeneity: it increased all NPCE indicators in the negative mismatch region but increased the NPA and CEA while reducing the NPY and CEY in the positive mismatch region. Moreover, cultivated land management scale and chemical fertilizer application intensity significantly mediated the relationship between the SMGPC and the NPCE. The findings offer guidance for optimizing grain production layout and promoting a green agricultural transformation.

## 1. Introduction

Grain is a fundamental strategic resource for human survival and development [1]. Food security, the cornerstone of poverty alleviation and sustainable development, has become a global research priority in recent decades [2]. As the world’s second most populous country, China’s domestic food security has long attracted considerable international attention [3]. The country has achieved remarkable progress in terms of grain production, feeding approximately 22% of the global population using only 7% of the world’s cultivated land [4]. Since the reform and opening-up, Chinese grain yield has increased 1.25-fold through an intensive agricultural model, which has caused a range of environmental problems, thereby jeopardizing sustainable agricultural development [5]. Notably, excessive and inefficient use of agrochemicals has triggered severe non-point source pollution and carbon emissions. Against the backdrop of China’s “dual carbon” goal and high-quality development strategy, the synergistic reduction in non-point source pollution from grain production (NP) and carbon emissions from grain production (CE) has become an essential pathway for the green transformation of agriculture [6]. Owing to regional disparities in resource endowments, economic development, urbanization, and population distribution, grain production and consumption are generally spatially mismatched at both the international and interprovincial scales [7,8]. This phenomenon is known as the spatial mismatch between grain production and consumption (SMGPC) [7]. In China, this spatial mismatch is particularly prominent at the interprovincial level, typified by the “north–to–south grain flow” pattern [9]. Since the 1990s, the SMGPC has become increasingly prominent in China, and interprovincial grain trade has emerged as a vital pathway for ensuring national food security [7]. However, this trade entails multiple challenges, including intensified resource and environmental pressures in major grain-producing regions and heightened supply security risks in major grain-consuming regions [10].

Previous studies have extensively explored the pattern of the SMGPC and its environmental impacts. Specifically, some scholars have identified an increasing spatial imbalance of provincial grain production and consumption in China during 2002–2012, marked by the growing distance between grain production and consumption gravity centers [11]. Moreover, the environmental impacts of grain trade originating from the SMGPC have attracted scholarly attention, with related studies focusing on three aspects. (1) The existing literature has demonstrated that China’s grain production–consumption imbalance drove virtual water flows with interregional grain transfer, thereby exacerbating water pressure and a gray water footprint in grain export areas [12]. Further research has revealed that virtual water flows embedded in China’s interprovincial grain trade increased national-level water loss and the water availability footprint [13,14]. (2) The intensified SMGPC has been proven to account for over 60% of the total growth in grain transport-related carbon emissions in China from 1990 to 2015 [15]. However, some scholars have discovered that interprovincial maize trade reduced national carbon emissions and nitrogen losses, with obvious regional differences: northeastern provinces showed great potential to curb both, whereas northwestern provinces increased total carbon emissions yet lowered the overall nitrogen losses by exporting maize [16]. (3) Some scholars demonstrated that developed economies imported cropland soil erosion from developing or agriculturally advanced economies via the global crop trade [17]. Other scholars have revealed that China’s SMGPC pattern harmed soil erosion control, with the embodied soil erosion from interprovincial grain trade induced by the SMGPC increasing 2.74-fold during 1990–2020 [7].

Although previous studies have provided valuable insights into the SMGPC and its environmental impacts, two research gaps remain. First, previous studies only characterized the SMGPC at the national level by comparing the distributions of grain production and consumption, failing to capture the evolutionary characteristics of provincial SMGPC. Thus, they cannot provide precise policy implications for regulating the grain production–consumption system and moderately restraining the SMGPC. Second, environmental impact assessments of the SMGPC relied on simulated interprovincial grain trade data, which may deviate from reality and did not evaluate the direction, magnitude, and underlying mechanisms of the SMGPC affecting non-point source pollution and carbon emissions from grain production (NPCE). Consequently, it is difficult for policymakers to formulate comprehensive and targeted NPCE reduction policies tailored to provincial SMGPC conditions.

Against this backdrop, this study focuses on three core objectives: (1) to reveal the spatiotemporal evolution of provincial SMGPC and NPCE in China; (2) to identify the impact of the SMGPC on the NPCE and its heterogeneity in different mismatch regions; and (3) to investigate the internal mechanism through which the SMGPC affects the NPCE. The possible innovations and potential contributions of this study are as follows:

First, it quantifies the provincial SMGPC using the spatial mismatch index and elucidates its spatiotemporal evolution, thus advancing existing macro-level analyses and providing guidance for policymakers to moderately control the SMGPC to ensure food security. Second, this study directly estimates the impact of the SMGPC on the NPCE using a multidimensional indicator system. Prior studies have generally assessed the environmental consequences of the SMGPC by analyzing virtual resources and ecological factor flows with interregional grain transfer, based on the simulation of interprovincial grain trade. This study used a fixed-effect model to examine both the impacts of the SMGPC on NPCE indicators—non-point source pollution per unit sown area (NPA), carbon emissions per unit sown area (CEA), non-point source pollution per unit yield (NPY), and carbon emissions per unit yield (CEY)—and the heterogeneity in these impacts across mismatch regions, thereby facilitating a multidimensional and precise investigation from both area-based and yield-based perspectives. This augments the current body of knowledge on the SMGPC and NPCE and provides guidance for NPCE reduction through moderating the SMGPC regulations. Third, this study explored the intrinsic mechanism through which the SMGPC affects the NPCE, focusing on the dual channels of the cultivated land management scale (CLMS) and chemical fertilizer application intensity (CFAI). The results of this study thus improve the general understanding of how the SMGPC affects the NPCE, enabling relevant authorities to integrate the regulations of the SMGPC, CLMS, and CFAI into a unified policy framework to reduce the NPCE more efficiently.

## 2. Theoretical Analysis

**Figure 1:**
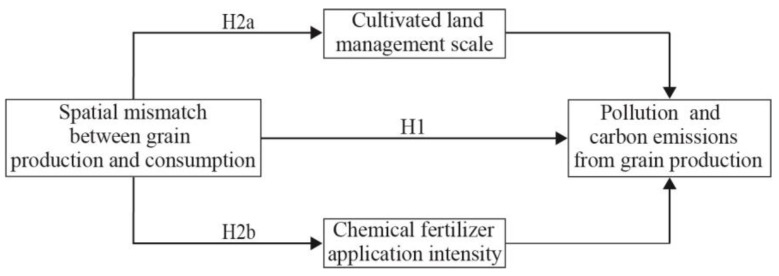
The mechanisms for the SMGPC affecting the NPCE.

The SMGPC reflects a structural imbalance between a region’s relative shares of national grain production and consumption. A higher SMGPC degree indicates a stronger systemic yield-increasing pressure. The positive mismatch region (production share > consumption share) faces exogenous pressure to boost grain yield and secure national grain supply, whereas the negative mismatch region (production share < consumption share) faces endogenous pressure to enhance local grain self-sufficiency. These pressures affect grain production practices and thus influence the NPCE mainly through three policy-supported channels: economic incentives, technological empowerment, and administrative regulations. The CLMS and CFAI are two core dimensions that reflect adjustments in grain production practices, representing the organization mode of production factors and the input intensity of chemical substances, respectively. These two types of behavioral variables are not only the most direct and sensitive response variables of local governments and farmland management entities under yield-increasing pressure but also serve as key mediating nodes linking the SMGPC to the NPCE. Therefore, the mediating roles of the CLMS and CFAI were further explored. Figure 1 presents the theoretical framework and research hypotheses of this study.

### 2.1. The Direct Impact of the SMGPC on the NPCE

The systemic yield-increasing pressure induced by the SMGPC affects the NPCE by influencing the grain-sown area, yield levels, and the associated NP and CE. In terms of economic incentives, China has introduced a set of agricultural subsidy programs (e.g., direct grain subsidies and comprehensive input subsidies) and fiscal reward programs (e.g., fiscal rewards for major grain-producing counties and land transfer) to increase farmers’ income and the grain output [18]. These economic incentives promote the expansion of the CLMS and grain-sown areas, an increase in agrochemical inputs, and the adoption of agricultural machinery [19,20]. While raising yields, these programs may also increase the NP and the CE from agrochemical and fossil fuel use [21]. Additionally, green agricultural subsidies show limited effectiveness due to high upfront costs and slow returns, thus hampering the willingness of farmers to adopt green practices [22]. Regarding technology empowerment, agricultural technology extension promotes the adoption of advanced technologies in grain production by reducing information asymmetry and adoption costs, thereby increasing the grain yield per unit area [23]. However, as rational economic agents, farmers often prioritize traditional agrochemical-intensive technologies based on cost–benefit trade-offs, which may increase the NP and the CE [24]. Moreover, some green agricultural technologies that rely on mechanized operations may inadvertently increase the CE [22]. With regard to administrative regulations, China has implemented a food security responsibility system since 1995, holding provincial authorities accountable [25]. This system devolves grain security responsibilities to local governments and incorporates them into political performance evaluations [26]. The system’s criteria—enhancing grain production capacity, implementing and improving grain support policies, and conserving farmland—drive local governments to adopt targeted measures to improve farmland quality and production conditions and incentivize grain production to boost yields [27]. However, this tends to induce high-input and high-output strategies, including excessive agrochemical use, marginal land cultivation, and unsustainable increases in the multiple cropping index [28]. These practices may directly increase the NP and the CE and indirectly increase them by requiring additional fertilizer inputs to maintain soil fertility [29].

Collectively, the systemic yield-increasing pressure from the SMGPC increases grain yield but may significantly increase the NP and the CE due to higher factor input intensity and improper farmland use. Notably, differences in resource endowments, policy support, and yield-increasing pressure between positive and negative mismatch regions may lead to heterogeneous impacts on the SMGPC and on the NPCE. Accordingly, hypothesis H1 was proposed:

**Hypothesis 1 (H1).**  *The SMGPC increases the overall NPCE; however, this effect may differ across mismatch regions.*

### 2.2. The Mechanism of the SMGPC Affecting the NPCE

The systemic yield-increasing pressure induced by the SMGPC may transmit its effects on the NPCE by influencing grain production practices, with the CLMS and the CFAI serving as critical mediating variables.

The SMGPC promotes CLMS expansion mainly through economic incentives and administrative regulations. Under economic incentives, direct grain subsidies and comprehensive input subsidies encourage farmers to expand their CLMS by boosting operational returns [30]. Additional subsidies, fiscal rewards, and policy-supported financial instruments for large-scale operators and land transfer (e.g., farmland mortgage and policy-oriented insurance) can lower the transaction costs and ease the credit constraints of land transfer and consolidation, thereby promoting CLMS expansion [31,32]. Under administrative regulations, local governments implement corresponding projects to fulfill targets of the food security responsibility system (e.g., the construction of high-standard farmland and the development of irrigation and water conservation infrastructure), which facilitates the integration of fragmented cultivated land and the improvement of cultivated land quality and agricultural production conditions [33]. This can help reduce cultivated land fragmentation, stabilize grain output, promote cultivated land transfer, and facilitate agricultural mechanization, all of which support CLMS expansion [34]. Moreover, fostering new grain production entities is a formal assessment metric that prompts fiscal and financial support, enabling these entities to access land, credit, and markets more effectively, thereby expanding the CLMS through land transfer [34,35]. Overall, the CLMS has a dual effect on the NPCE, with the inhibitory effect dominant. On the one hand, an expanded CLMS facilitates the optimization of factor allocation and the adoption of precision agriculture technologies, thereby reducing agrochemical inputs and the associated NP and CE [36,37]. Large-scale operators also possess stronger financial capacity, information access, and risk tolerance, and are more oriented toward long-term returns. Combined with the lower application costs of green technologies at larger operational scales, these factors enhance their willingness and ability to adopt green practices, further curbing the NP and the CE [38,39]. On the other hand, CLMS expansion increases labor demand. Amid agricultural labor out-migration and rising wages, large-scale operators tend to substitute labor with machinery and agrochemicals, thereby increasing the NP and the CE [40]. Therefore, hypothesis H2a was formulated:

**Hypothesis 2a (H2a).**  *The SMGPC influences the NPCE through the CLMS.*

The SMGPC increases CFAI mainly through three channels. First, direct grain subsidies and chemical fertilizer input subsidies reduce the relative price of chemical fertilizers, thereby motivating the increase in CFAI by lowering input costs and boosting returns from grain production [41]. Second, to achieve a rapid increase in grain output, farmers, as rational economic agents, tend to choose traditional fertilizer-intensive technologies with low upfront investment costs and rapid returns, resulting in a technological lock-in effect [42]. In contrast, green fertilization technologies are difficult to popularize due to high initial investment and long payback periods, reinforcing the path dependence on chemical fertilizers [43]. Third, the grain security responsibility system quantifies local production targets and imposes institutional pressure, thereby encouraging input-intensive strategies. This further drives CFAI directly through land-use adjustments (e.g., cultivating marginal land and raising multiple cropping index) that require more chemical fertilizer to maintain soil fertility and productivity [28]. A moderate increase in CFAI enhances grain yield per unit area by improving soil fertility [44]. However, as a major source of the NP and the CE, rising CFAI directly increases them and may restrain yield growth by causing soil degradation and groundwater pollution [45,46]. Accordingly, hypothesis H2b was formulated:

**Hypothesis 2b (H2b).**  *The SMGPC influences the NPCE through CFAI.*

## 3. Methods and Data

### 3.1. Methods

#### 3.1.1. Quantification of the SMGPC

The spatial mismatch index, which is widely used to measure the spatial imbalance between interrelated factors, was adopted to quantify the SMGPC [47,48]. Consistent with China’s food security priorities, grain in this study refers to cereals, legumes, and tubers. The SMGPC was calculated as follows:

(1) $$SMGPC_{i}=\frac{1}{C}(\frac{P_{i}}{P}C-C_{i})\times 100$$

(2) $$W_{i}=\left|SMGPC_{i}\right|/\sum_{i=1}^{n}\left|SMGPC_{i}\right|$$

where *SMGPC_i_* is the spatial mismatch index of grain production and consumption for province *i*; *P* and *C* represent the national grain production and consumption, respectively; *P_i_* and *C_i_* denote the grain production and consumption of province *i*, respectively; |*SMGPC_i_*| is the absolute value of *SMGPC_i_*, which refers to the SMGPC degree for province *i*; and *W_i_* is the contribution degree of the SMGPC degree for province *i*. *SMGPC_i_* > 0 indicates a positive mismatch, while *SMGPC_i_* < 0 indicates a negative mismatch.

#### 3.1.2. Baseline Regression Model

This study used a fixed-effect model to explore the impact of the SMGPC on the NPCE. To account for time lags in farmland-use adjustments and mitigate potential endogeneity issues, the one-period lagged SMGPC degree was selected as the explanatory variable. The baseline regression model was set as follows:

(3) $$NPCE_{it}=\alpha_{0}+\alpha_{1}\left|SMGPC_{i,t-1}\right|+\phi_{0}X_{it}+\mu_{i}+\omega_{t}+\varepsilon_{it}$$

where *NPCE_it_* represents the NPCE of province *i* in year *t*; |*SMGPC*_*i*,*t*−1_| is the SMGPC degree of province *i* in year *t* − 1; *X_it_* denotes the control variables; *α*_0_ stands for the constant term; *α*_1_ and *ϕ*_0_ refer to the coefficients to be estimated; *μ_i_* and *ω_t_* are the individual and time fixed effects, respectively; and *ɛ_it_* indicates the random error term.

#### 3.1.3. Mediating Effect Model

A two-step mediating effect model was adopted to examine the internal mechanism of the SMGPC affecting the NPCE [49]. The CLMS and CFAI were tested as potential mediating variables using the following formulas:

(4) $$M_{it}=\beta_{0}+\beta_{1}\left|SMGPC_{i,t-1}\right|+\beta_{2}X_{it}+\mu_{i}+\omega_{t}+\varepsilon_{it}$$

(5) $$IPCE_{it}=\lambda_{0}+\lambda_{1}\left|SMGPC_{i,t-1}\right|+\lambda_{2}M_{it}+\lambda_{3}X_{it}+\mu_{i}+\omega_{t}+\varepsilon_{it}$$

where *M_it_* denotes the mediating variable for province *i* in year *t*; *β*_0_ and *λ*_0_ stand for the constant term; and *β*_1_, *β*_2_ and *λ*_1_–*λ*_3_ are the coefficients to be estimated. The remaining variables are identical to those specified in Equation (3).

Based on the above model, the mediating effects of the CLMS and CFAI were tested in three steps: (1) examine the statistical significance of *β*_1_ and *λ*_2_. If both were significant, a mediating effect was confirmed. (2) Test the significance of *λ*_1_. If *λ*_1_ was insignificant, it indicated a full mediating effect; otherwise, step 3 was applied. (3) Compare the signs of *β*_1_*λ*_2_ and *λ*_1_. If they had the same signs, there was a partial mediating effect; if they had opposite signs, there was a suppression effect.

### 3.2. Variable

#### 3.2.1. Core Explained Variable

The NPCE was the core explained variable measured from two complementary dimensions: per unit sown area (NPA and CEA) and per unit yield (NPY and CEY). The NPA and CEA were defined as the ratio of the total equal-standard pollution load of the NP (quantified in Supplementary S2) and the total CE (measured in Supplementary S3) to the total grain-sown area, respectively, reflecting the environmental pressure of cultivated land for grain production. The NPY and CEY were calculated as the ratio of the total equal-standard pollution load of the NP and the total CE to the total grain yield, respectively, reflecting the environmental costs arising from grain production.

#### 3.2.2. Explanatory Variable

The SMGPC degree (|SMGPC|) was the core explanatory variable. As the systemic yield-increasing pressure induced by the SMGPC requires a full production cycle to translate into adjustments in grain production practices, its impact on the NPCE has a time lag.

#### 3.2.3. Mediating Variables

The CLMS and CFAI were the mediating variables. The CLMS was measured by the ratio of cultivated land area to agricultural labor force, reflecting the operation scale of cultivated land [21]. The CFAI was defined as the ratio of the amount of chemical fertilizer applied to the total crop-sown area, representing the input level of chemical fertilizers [50].

#### 3.2.4. Control Variables

Referring to prior studies, we selected the following control variables to account for their potential influence on the NPCE: (1) multiple cropping index (MCI), measured by the ratio of the total crop-sown area to the cultivated land area [6,21,51]; (2) agricultural disaster rate (ADR), assessed by the proportion of the affected area of crops to the total crop-sown area [6,21,51]; (3) financial support for agriculture (FSA), characterized by the share of agricultural expenditure in the total fiscal expenditure [6,21,51]; (4) education level in rural areas (ELRA), proxied by the average years of schooling of rural residents [6,21,51]; (5) urbanization rate (UR), determined by the proportion of the urban population to the total population [6,21,51]; and (6) economic development level (PGDP), measured by gross domestic product per capita [6,21,51].

### 3.3. Study Areas and Data Sources

**Figure 2:**
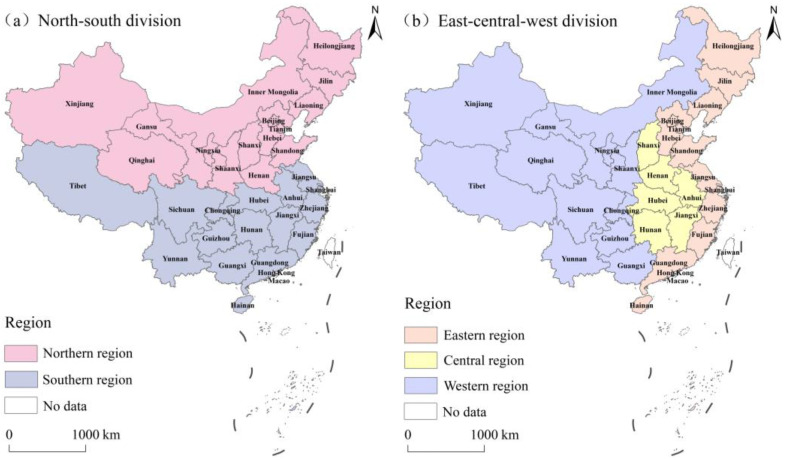
The study area and regional divisions.

This study used panel data of 31 provinces in mainland China during 2000–2023. To capture the regional heterogeneity in the SMGPC and the NPCE, two classification schemes were adopted (Figure 2). Provinces were divided into the northern and southern regions for the SMGPC analysis [52] and into the eastern, central, and western regions for the NPCE analysis [53]. Data on agricultural production and factor inputs were obtained from the China Rural Statistical Yearbook. Socioeconomic indicators were collected from the China Statistical Yearbook. Industrial product data was derived from the China Light Industry and Food Industry Yearbooks. Rural educational level was sourced from the China Population and Employment Statistics Yearbook. Missing values were supplemented using provincial statistical yearbooks.

## 4. Results

### 4.1. Spatiotemporal Evolution of the SMGPC

#### 4.1.1. Temporal Evolution of the SMGPC

**Figure 3:**
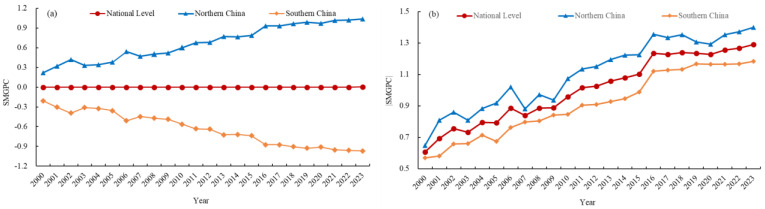
The temporal evolution of the SMGPC (**a**) and the SMGPC degree (**b**) in China, 2000–2023.

Figure 3 presents the temporal variation in the SMGPC and SMGPC degree (|SMGPC|) in China from 2000 to 2023. Nationally, the average SMGPC remained consistently close to 0, whereas the average SMGPC degree increased from 0.61 to 1.29, indicating a continuous intensification of overall spatial mismatch. In the northern region, the average SMGPC and the average SMGPC degree increased from 0.22 and 0.65 to 1.04 and 1.40, respectively, demonstrating a strengthening positive mismatch. Conversely, in the southern region, the average SMGPC decreased from −0.21 to −0.97, and the average SMGPC degree increased from 0.57 to 1.18, reflecting a deepening negative mismatch.

#### 4.1.2. Spatial Pattern of the SMGPC

**Figure 4:**
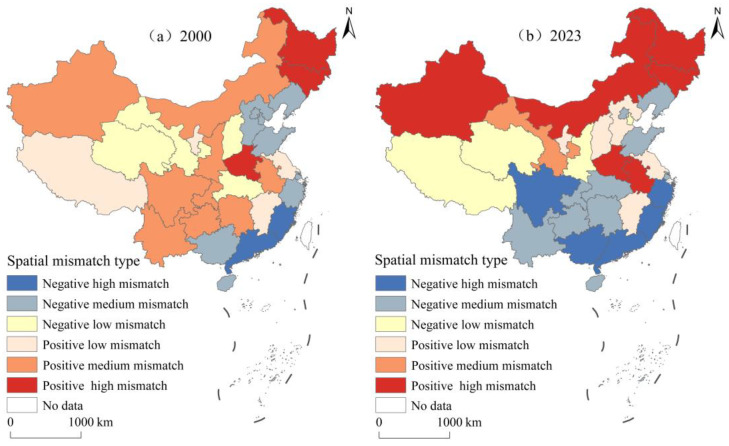
The spatial pattern of the SMGPC in China, 2000–2023.

According to previous studies [8], this study categorized the SMGPC into six types. Figure 4 shows the spatial pattern of the SMGPC in China. During 2000–20 the SMGPC exhibited three core characteristics: expanding negative mismatch, intensifying spatial polarization, and prominent regional differentiation. Specifically, the proportion of negative mismatch provinces increased from 48.39% to 61.29%. In the positive mismatch region, the polarization trend intensified: the proportion of provinces with positive high mismatch and positive low mismatch rose from 9.68% and 12.90% to 19.35% and 16.13%, respectively, whereas the proportions of provinces with positive medium mismatch decreased from 29.03% to 3.23%. These changes further confirm the concentration of grain production in core producing areas. Additionally, the regional differences between the northern and southern regions were prominent. In the southern region, the proportion of negative mismatch provinces increased from 43.75% in 2000 to 81.25% in 2023, reflecting a significant weakening of the grain self-sufficiency capacity. Conversely, the proportion of positive mismatch provinces in the northern region increased from 46.67% to 60%, consolidating its dominant role in grain supply.

### 4.2. Spatiotemporal Evolution of the NPCE

#### 4.2.1. Temporal Evolution of the NPCE

**Figure 5:**
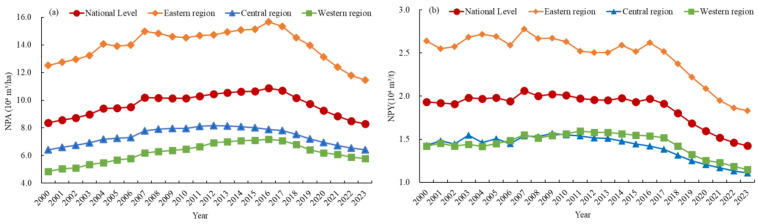
The temporal evolution of NPA (**a**) and NPY (**b**) in China, 2000–2023.

Figure 5 presents the temporal evolution of the average NPA and NPY in China from 2000 to 2023. As shown in Figure 5a, the national NPA first increased and then decreased. It first increased from 8.36 × 10^4^ m^3^/ha in 2000 to a peak of 10.87 × 10^4^ m^3^/ha in 2016 and then decreased continuously to 8.29 × 10^4^ m^3^/ha in 2023, with a cumulative net decrease of 0.89% over the study period. Regionally, the eastern, central, and western regions followed this trend, with the eastern region maintaining the highest NPA throughout the study period, followed by the central and western regions. Among the three regions, only the eastern region exhibited a net decrease in NPA (8.36%). Figure 5b shows that the national NPY fluctuated around 2.00 × 10^4^ m^3^/t during 2000–2016 and then continued to decrease to 1.43 × 10^4^ m^3^/t in 2023, with a cumulative net decrease of 27.49% over the study period. Regionally, the NPY in the eastern, central, and western regions decreased with fluctuations, with the eastern region having the highest absolute level and the largest net reduction (28.76%). Chow tests confirm 2016 as a significant structural break for NPA and NPY (Table S3), and the continuous decline of both indicators after 2016 indicates the effectiveness of the targeted policies for controlling NP.

**Figure 6:**
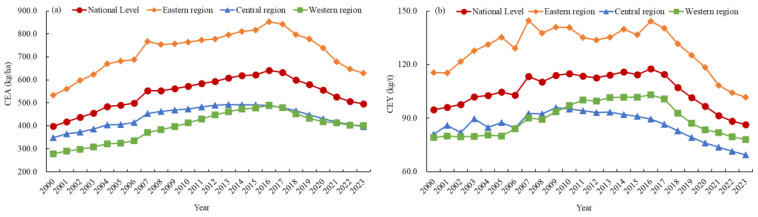
The temporal evolution of CEA (**a**) and CEY (**b**) in China, 2000–2023.

Figure 6 shows the temporal evolution of the average CEA and CEY in China from 2000 to 2023. As shown in Figure 6a, the national CEA first rose and then dropped, rising from 398.86 kg/ha in 2000 to a peak of 642.02 kg/ha in 2016 and then dropping steadily to 495.77 kg/ha in 2023, with a cumulative net increase of 24.30% during the study period. Regionally, the eastern, central, and western regions exhibited this trend, with the eastern region maintaining the highest CEA throughout the study period, followed by the central and western regions. During the study period, the average CEA in the eastern, central, and western regions increased by 17.97%, 13.81%, and 43.97%, respectively. Figure 6b shows that the national CEY increased with fluctuations from 94.77 kg/t in 2000 to a peak of 117.75 kg/t in 2016, and then decreased consistently to 86.34 kg/t in 2023, with a cumulative net decrease of 8.90% over the study period. Regionally, the CEY in the eastern, central, and western regions fluctuated before 2016 and declined steadily thereafter, with the eastern region having the highest CEY throughout the study period. Chow tests confirm 2016 as a significant structural break for CEA and CEY (Table S3), and the continuous downward trend of both indicators after 2016 confirms the extensive effectiveness of targeted policies for promoting the low-carbon transformation of agriculture.

#### 4.2.2. Spatial Patterns of the NPCE

**Figure 7:**
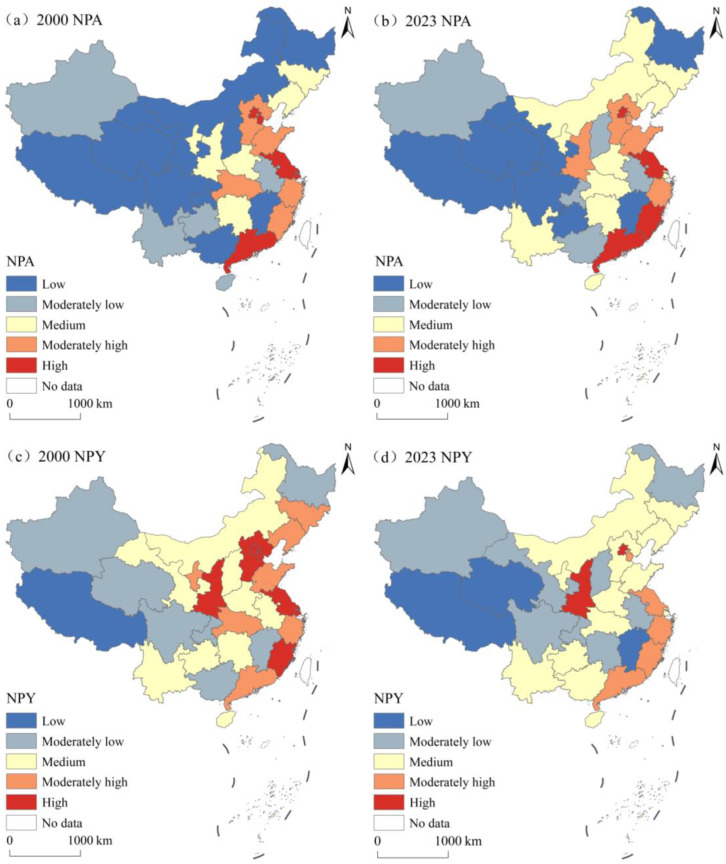
The spatial mismatch patterns of NPA and NPY in China, 2000–2023.

All four NPCE indicators were categorized into five grades using the natural breakpoint method. The spatial patterns of NPA and NPY in 2000 and 2023 are shown in Figure 7. Both NPA and NPY exhibited a persistent spatial pattern of “high in the eastern region, low in the central and western regions”, but their grade structures and high-value agglomerations differed. The grade structure of NPA underwent significant changes: the proportions of low-grade and high-grade provinces decreased, whereas those of medium-grade and moderately high-grade provinces increased; the proportion of moderately low-grade provinces remained stable. Specifically, the proportions of low-grade and high-grade provinces decreased from 32.26% and 16.13% to 22.58% and 12.90%, respectively, whereas the total proportion of moderately low-grade, medium-grade, and moderately high-grade provinces increased from 51.61% to 64.52%. In 2000, high-grade provinces were concentrated in the eastern municipalities and coastal areas, forming a contiguous high-value belt. By 2023, this concentration area had shrunk slightly. Meanwhile, the three western provinces that originally belonged to the low grade rose to higher grades, but the east–west disparity remained unchanged. The grade distribution of NPY shifted markedly: the proportion of medium-grade provinces surged from 29.03% to 41.94%, becoming the largest category by 2023, whereas that of low-grade provinces increased from 3.23% to 9.68%, and that of high-grade provinces decreased sharply from 22.58% to 6.45%. Overall, NPY grades shifted toward the median values, with a slight expansion of the lowest category. In 2000, high-grade provinces were concentrated in the eastern municipalities and coastal areas, forming scattered agglomerations. By 2023, most high-grade provinces in 2000 had shifted to lower grades, shrinking the highest-grade cluster from seven to two.

**Figure 8:**
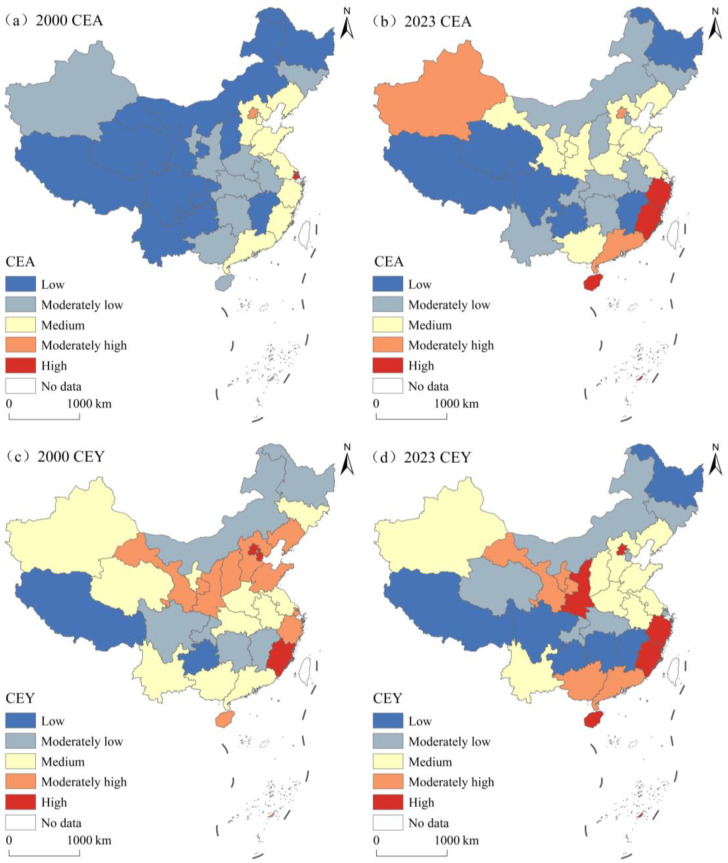
The spatial mismatch patterns of CEA and CEY in China, 2000–2023.

The spatial patterns of CEA and CEY in 2000 and 2023 are shown in Figure 8. Both CEA and CEY maintained a consistent spatial pattern of “high in the eastern region, low in the central and western regions” during the study period, but their grade structures and high-value agglomerations differed. The grade structure of CEA changed substantially: the proportions of low-grade and moderately low-grade provinces decreased significantly, whereas medium-grade, moderately high-grade, and high-grade provinces increased significantly, presenting an overall trend of transitioning to higher grades. Specifically, the proportion of low-grade provinces decreased from 35.48% to 19.35%, the proportion of high-grade provinces increased from 3.23% to 9.68%, and the total proportion of moderately low-grade, medium-grade, and moderately high-grade provinces increased from 61.29% to 70.97%. In 2000, moderately high-grade and high-grade provinces were scattered in eastern municipalities. By 2023, the number of high-grade provinces increased from one to three. The grade structure of CEY shifted markedly: the proportion of low-grade and moderately low-grade provinces increased significantly, whereas the proportions of medium-grade and moderately high-grade provinces decreased significantly, reflecting a shift toward lower grades. Specifically, the proportions of low-grade, moderately low-grade, and high-grade provinces increased from 6.45%, 19.35%, and 9.68% to 16.13%, 32.26%, and 16.13%, respectively, whereas the total proportion of medium-grade and moderately high-grade provinces decreased from 64.52% to 35.48%. In 2000, moderately high-grade and high-grade provinces were concentrated in the eastern region. By 2023, the high-value agglomeration of CEY expanded toward the eastern coastal region.

### 4.3. The Impact of the SMGPC on the NPCE

#### 4.3.1. Baseline Regression Results

Table 1 and Table 2 report the regression results for area-scaled (NPA, CEA) and yield-scaled (NPY, CEY) indicators, respectively. The F-test and Hausman test results for Models (1)–(6) in both tables are significant at the 1% level. Therefore, the two-way fixed-effect model was adopted for all baseline regressions.

As shown in Models (1) and (2) in Table 1, the coefficients of |SMGPC| were 0.049 and 0.046, respectively (*p* < 0.01 for each). This suggests that the increased SMGPC degree significantly increased NPA and CEA nationwide. Models (3) and (4) show that in the positive mismatch region, the coefficients of |SMGPC| were 0.045 and 0.030, respectively (*p* < 0.1 for each). Meanwhile, in the negative mismatch region, the coefficients of |SMGPC| in Models (5) and (6) were 0.108 and 0.124, respectively (*p* < 0.01 for each). These results reveal that the increased SMGPC degree drove NPA and CEA in both the positive and negative mismatch regions, with a stronger effect in the negative mismatch region. These findings confirm that SMGPC exacerbated the environmental pressure on cultivated land for grain production at national and regional levels. Control variables showed consistent and heterogeneous effects on NPA and CEA: the MCI negatively affected both NPA and CEA across all samples; the ADR was not correlated with NPA and CEA across all samples; FSA positively affected both NPA and CEA across all samples; the ELRA negatively affected NPA nationwide and in the negative mismatch region, while positively affecting NPA in the positive mismatch region and CEA nationwide and in the positive mismatch region; the UR was negatively associated with both NPA and CEA nationwide and in the negative mismatch region, and with NPA in the positive mismatch region; and the PGDP was positively related to both NPA and CEA across the three samples.

As illustrated in Models (1) and (2) in Table 2, the coefficients of |SMGPC| for NPY and CEY were 0.017 and 0.014 (*p* < 0.1 for each), respectively. This indicates that the increased SMGPC degree significantly drove NPY and CEY nationwide. Notably, there is a distinct directional heterogeneity in the effect of the SMGPC degree on NPY and CEY between the positive and negative mismatch regions. Models (3) and (4) demonstrate that in the positive mismatch region, the coefficients of |SMGPC| for NPY and CEY are −0.020 (*p* < 0.05) and −0.035 (*p* < 0.01), respectively. Conversely, Models (5) and (6) show that in the negative mismatch region, the coefficients of |SMGPC| for NPY and CEY are 0.133 and 0.149, respectively (*p* < 0.01 for each). These findings reveal that the SMGPC increased the environmental costs of grain production nationwide and in the negative mismatch region but lowered them in the positive mismatch region. Control variables exerted both consistent and heterogeneous impacts on NPY and CEY: the MCI negatively affected both NPY and CEY nationwide and in the negative mismatch region, and CEY in the positive mismatch region; the ADR was positively associated with both NPY and CEY across all samples; FSA positively affected both NPY and CEY across all samples; the ELRA showed a negative correlation with NPY nationwide and in the negative mismatch region, and with CEY in the negative mismatch region, but a positive correlation with CEY in the positive mismatch region; the UR negatively influenced NPY and CEY across all samples; and the PGDP was positively associated with both NPY and CEY across all samples.

Collectively, the SMGPC degree significantly affected all four NPCE indicators, with obvious heterogeneity in different mismatch regions. Therefore, hypothesis H1 was validated.

#### 4.3.2. Endogeneity and Robustness Test Results

First, we conducted the endogeneity test using the instrumental variable method. To solve the problem of endogeneity, we used the average of the one-period lag values of the SMGPC across adjacent provinces as the instrumental variable for regression. Second, we excluded the atypical samples. Considering the distinct economic structure and limited role in grain production of China’s four municipalities (Beijing, Tianjin, Shanghai, and Chongqing), we conducted the regression again by excluding them. Third, we shortened the panel data duration. To address the potential impact of 2006 agricultural policy adjustments—including the abolition of agricultural tax and the introduction of agricultural subsidies—which may influence the NPCE, we shortened the panel data period to 2006–2023 and re-conducted the regression. Fourth, we employed a Tobit model to examine the sensitivity of the baseline findings to the model specification. Fifth, we adopted an alternative grouping criterion by excluding provinces with negligible spatial mismatch (|SMGPC| < 0.1) to test the sensitivity of the baseline regression results to the discrete classification threshold. Tables S4–S8 show that the effects of the SMGPC on NPA, CEA, NPY, and CEY were consistent with the baseline regression results, which confirms the reliability and robustness of the baseline findings.

#### 4.3.3. Mechanism Test Results

Table 3 and Table 4 report the mediating effects of the CLMS and CFAI, respectively. Model (1) in Table 3 shows that increasing the SMGPC degree significantly promoted CLMS expansion nationwide and in both positive and negative mismatch regions. Models (2)–(5) reveal that CLMS expansion significantly reduced all four NPCE indicators (NPA, CEA, NPY, and CEY) across all samples. After accounting for the CLMS, the direct effect of the SMGPC degree varied by region. Nationally and in the negative mismatch region, the SMGPC degree retained a significant positive direct effect on all four NPCE indicators. In the positive mismatch region, the direct effect of the SMGPC degree was heterogeneous: it remained positive for NPA and CEA but turned significantly negative for NPY and CEY. Collectively, the CLMS significantly mediated the effect of the SMGPC degree on the four NPCE indicators. However, the role of the CLMS varied by region. Nationally and in the negative mismatch region, CLMS expansion significantly suppressed the impact of the SMGPC degree on all four NPCE indicators. In the positive mismatch region, CLMS expansion significantly suppressed the effects of the SMGPC degree on NPA and CEA, whereas it partially mediated the effects of the SMGPC degree on NPY and CEY. Therefore, H2a was confirmed.

Model (1) in Table 4 shows that the increased SMGPC degree significantly promoted the rise in CFAI across all samples, and Models (2)–(5) indicate that the CFAI positively affected all four NPCE indicators. The direct effect of the SMGPC degree after controlling for the CFAI varied by regions. Nationally, the SMGPC degree retained a significantly positive direct effect on NPA but not on CEA, NPY, or CEY, indicating that the CFAI partially mediated the relationship between the SMGPC degree and NPA, while fully mediating the relationship between the SMGPC degree and the other three indicators. In the positive mismatch region, the direct effect of the SMGPC degree remained significantly positive for NPA and negative for NPY and CEY, but insignificant for CEA, confirming the CFAI as a partial mediator between the SMGPC degree and NPA, a full mediator between the SMGPC degree and CEA, and a suppressor between the SMGPC degree and NPY and CEY. In the negative mismatch region, the SMGPC degree retained significantly positive direct effects on CEA, NPY, and CEY but not on NPA, demonstrating that the CFAI was a full mediating variable between the SMGPC degree and NPA and a partial mediating variable between the SMGPC degree and the other three indicators. In summary, the CFAI significantly mediated the impact of the SMGPC degree on the four NPCE indicators, with notable regional differences in the mediating mechanisms. Thus, H2b was supported.

## 5. Discussion

### 5.1. Intensification and Regional Polarization of the SMGPC

This study confirmed that there is a pronounced and intensifying SMGPC in China, reflecting an uneven spatial restructuring of the grain production–consumption system. The increased SMGPC degree, along with the increasing proportions of provinces with negative mismatch and provinces with high positive mismatch, indicates that grain production has further concentrated in the core producing areas in the northern region, and the southern region has become more dependent on the grain supplies from the northern region. This is consistent with the findings of [54], who found a northward agglomeration of grain output, intensified spatial mismatch between grain output and demand, and a deteriorating supply–demand relationship in the major sales areas in China during 2000–2022. Moreover, the contribution degree of the provincial SMGPC degree was highly polarized: eight high-contributing provinces—including major grain producers (Heilongjiang, Inner Mongolia, Jilin, and Henan) and economically developed coastal consumers (Guangdong, Zhejiang, and Fujian)—accounted for 71.31% of the national total in 2023. This pattern is consistent with the simulated interprovincial grain trade network reported by [7], which showed that in 2020, four major grain exporting provinces (Heilongjiang, Jilin, Inner Mongolia, and Henan) accounted for 82.09% of interprovincial grain exports, while four major importing provinces (Zhejiang, Fujian, Guangdong, and Guizhou) consumed 42.96% of the interprovincial imported grains. Coordinating the grain production and consumption in these key provinces is vital to moderately curbing the intensification of the SMGPC.

### 5.2. Divergent Trends and Spatial Disparities in the NPCE Evolution

This study revealed divergent temporal trends and obvious spatial disparities in the NPCE indicators, associated with the transformation of agricultural production modes and spatial heterogeneity in production factor input and utilization efficiency. Temporally, all four NPCE indicators (NPA, NPY, CEA, and CEY) followed a consistent pattern, with 2016 as the turning point: increasing fluctuations before 2016 due to intensive agrochemical inputs and declining continuously thereafter, driven by China’s agricultural green transformation policies, including the zero-growth action of chemical fertilizers and pesticides (2015) and agricultural support and protection subsidies (2016). This pattern is similar to existing studies: [6] identified an inverted “U” trend in the average coupling degree of agricultural pollution and carbon emissions in China during 2001–2021, with the peak occurring in 2014, and [55] verified that the “Two Zero” policy significantly reduced the application of chemical fertilizers and pesticides in China during 2015–2022. Notably, the national NPA and CEA exhibited a net increase, whereas the national NPY and CEY showed a net decrease. This reflects the increased environmental pressure per unit sown area but reduced environmental cost per unit yield, attributed to improved land productivity. All four NPCE indicators showed a spatial pattern of “high in the eastern region, low in the central and western regions”, stemming from east–west differences in factor input intensity, production conditions, and production efficiency.

### 5.3. Heterogeneous Impacts of the SMGPC on the NPCE

This study found that the SMGPC exerted significant heterogeneous impacts on the NPCE across mismatch regions. Nationally, the increase in the SMGPC degree generally drove all four NPCE indicators (NPA, NPY, CEA, and CEY), confirming that the SMGPC significantly increased environmental pressure and costs. This suggests that the systemic yield-increasing pressure from the SMGPC may encourage extensive factor input and farmland use practices to secure grain supply, thereby raising the NPCE indicators [29,56]. However, these impacts diverged by mismatch region: in the negative mismatch region, the increasing SMGPC degree drove all four NPCE indicators, whereas in the positive mismatch region, it increased NPA and CEA but reduced NPY and CEY. This difference may stem from the higher grain output efficiency in positive-mismatch provinces, where the growth rate of grain yield per unit sown area outpaced that of the NP and the CE per unit sown area induced by the SMGPC, thereby reducing NPY and CEY. In contrast, negative-mismatch provinces may rely on extensive factor inputs to ensure grain output but exhibit relatively slow yield growth, collectively leading to higher environmental pressures and costs. These findings support [57], who reported that during 1997–2020, the grain yield grew faster than carbon emissions from the grain production in major grain-producing regions in China, while grain-yield growth lagged behind that of carbon emissions in major grain-consuming regions and grain production–consumption balance regions, and those regions still relied heavily on extensive factor inputs.

### 5.4. Transmission Mechanisms: The CLMS and CFAI as Core Mediating Channels

The mechanism test results revealed that the SMGPC affected the NPCE through the CLMS and CFAI. The increased SMGPC degree significantly drove the expansion of the CLMS and the increase in the CFAI across all samples, indicating that the systemic yield-increasing pressure originating from the SMGPC may facilitate adjustments in grain production practices via administrative and economic measures. The CLMS expansion significantly reduced all four NPCE indicators across all samples, suggesting that CLMS expansion could improve factor allocation efficiency and reduce agrochemical inputs, thereby enhancing grain production capacity while mitigating the NP and the CE. Our findings align with [58], who found that large-scale operations could promote the synergistic reduction in agricultural non-point source pollution and carbon emissions in China’s major grain-producing areas. Moreover, we found that an increased CFAI significantly enhanced all four NPCE indicators, indicating that while chemical fertilizer input boosted grain yield, it drove faster growth in the NP and the CE.

### 5.5. Limitations and Prospects

This study has several limitations that need to be considered in future research. First, provincial grain consumption was estimated using grain conversion coefficients due to the absence of official data, failing to adequately account for the regional heterogeneity in these conversion coefficients and affecting the accuracy of the SMGPC quantification. Future research should incorporate micro-survey data to calibrate the estimates and improve the accuracy of the SMGPC measurement. Second, owing to the classification of research samples by mismatch types, the data for each subgroup constituted an unbalanced panel, failing to satisfy the sample continuity requirements for spatial econometric models. Therefore, this study employed a fixed-effect model to explore the effect of the SMGPC on the NPCE, excluding the spillover effect of the SMGPC. Third, the mechanism test results show that the transmission mechanisms of the SMGPC affecting the NPCE are complex. Therefore, other potential mediating variables may exist. Future research could incorporate additional mediating variables to gain a more complete understanding of the underlying transmission mechanisms.

## 6. Conclusions and Policy Implications

### 6.1. Conclusions

Based on panel data of 31 provinces in China from 2000 to 2023, this study examined the impact of the SMGPC on the NPCE using fixed-effect and mediating effect models. The main conclusions are as follows:

(1) China’s SMGPC intensified markedly during 2000–2023. The average SMGPC degree (|SMGPC|) increased from 0.61 to 1.29, and the proportion of negative mismatch provinces increased from 48.39% to 61.29%. In the northern region, the positive mismatch strengthened, and the share of positive mismatch provinces increased from 46.67% to 60%. In the southern region, the negative mismatch significantly deepened, and the proportion of negative-mismatch provinces increased from 43.75% to 81.25%.

(2) All four NPCE indicators (NPA, CEA, NPY, and CEY) peaked in 2016 and then declined continuously. From 2000 to 2023, the national NPA, NPY, and CEY decreased by 0.89%, 27.49%, and 8.90%, respectively, while the national CEA increased by 24.30%. All four NPCE indicators exhibited a persistent spatial pattern of “high in the eastern region, low in the central and western regions”, but their high-value agglomerations differed: high NPA and CEA formed a contiguous eastern coastal belt, while high NPY and CEY appeared as scattered clusters.

(3) The SMGPC exerted significant and heterogeneous impacts on the NPCE. Nationally, the increased SMGPC degree significantly drove all four NPCE indicators. Regionally, it increased all four NPCE indicators in the negative mismatch region, but increased NPA and CEA while reducing NPY and CEY in the positive mismatch region.

(4) The SMGPC degree affected the NPCE by enhancing the CLMS and CFAI. However, their mediating roles differed: the CLMS expansion reduced all four NPCE indicators, whereas an increased CFAI drove them. These opposing effects, together with the regional heterogeneity of SMGPC’s direct impacts on the four NPCE indicators, led to the heterogeneous mediating effects of the CLMS and CFAI across mismatch regions.

### 6.2. Policy Implications

(1) To ensure national food security, policymakers should systematically optimize the grain production layout and enhance the grain production capacity of consumption-dependent regions to moderately constrain the continuous intensification of SMGPC. A dual evaluation system integrating grain production-consumption matching and resource-environmental carrying capacity should be established to guide the formulation of a national medium and long-term grain production plan. Meanwhile, the negative mismatch region should enforce farmland protection policies, revitalize abandoned farmland, and improve farmland quality and agricultural production conditions to enhance farmland productivity and regional grain self-sufficiency. 

(2) Differentiated NPCE reduction strategies need to be implemented to achieve synergy between food security and environmental sustainability. The eastern region, with the highest NPCE and fastest decline, should transition from the current “high-input, high-output” production model to a “high-efficiency, low-emission” one by reinforcing agrochemical supervision, promoting precision agriculture, and incorporating NPCE reduction into the performance assessment of local governments. The western region, with the fastest growth in NPA, CEA, and CEY, should enhance resource use efficiency and curb agrochemical overuse to avoid falling into the “high-consumption, high-pollution” development trap. The central region, where NPCE indicators are at moderate to low levels, should stabilize grain output and enhance production efficiency by popularizing green agricultural technologies to simultaneously increase grain yield while reducing the NP and the CE.

(3) Tailored governance measures should be formulated for different mismatch regions, considering the heterogeneous impact of the SMGPC on the NPCE. For positive-mismatch provinces, their grain production advantages should be further leveraged through technological innovation and adoption to safeguard national food security and amplify the inhibitory effect of the SMGPC degree on NPY and CEY. Meanwhile, they should optimize resource allocation, reduce agrochemical dependence, and promote green and low-carbon farming practices to alleviate the increase in NPA and CEA caused by the SMGPC. For negative mismatch provinces, it is necessary to enforce standardized agrochemical inputs, increase green factor inputs, popularize high-efficiency and low-emission agricultural technologies, and incorporate NPCE indicators into agricultural development assessments to curb the promoting effect of the SMGPC degree on the four NPCE indicators while increasing grain output.

(4) Considering that the SMGPC degree enhanced the CLMS and CFAI, whereas the CLMS expansion reduced the four NPCE indicators, and the increase in CFAI drove them, authorities should regulate these two mediating channels. Specifically, targeted measures should be taken to accelerate the moderate expansion of the CLMS by facilitating land transfer, reducing the costs of large-scale operations, and consolidating fragmented plots. Moreover, it is imperative to strictly control the CFAI and reduce the overuse of chemical fertilizers by popularizing soil testing and formulated fertilization, substituting green manure and organic fertilizers with chemical fertilizers, and establishing regulatory mechanisms for chemical fertilizer inputs.

## Figures and Tables

**Table 1:** Baseline regression results of the SMGPC on NPA and CEA.

Variables	Overall		Positive Mismatch Region		Negative Mismatch Region	
	ln*NPA*	ln*CEA*	ln*NPA*	ln*CEA*	ln*NPA*	ln*CEA*
	(1)	(2)	(3)	(4)	(5)	(6)
*|SMGPC|*	0.049 ***	0.046 ***	0.045 ***	0.030 ***	0.108 ***	0.124 ***
	(0.0094)	(0.0103)	(0.0095)	(0.0107)	(0.0205)	(0.0215)
*MCI*	−0.345 ***	−0.416 ***	−0.215 ***	−0.432 ***	−0.355 ***	−0.414 ***
	(0.0315)	(0.0343)	(0.0660)	(0.0745)	(0.0393)	(0.0413)
*ADR*	−0.0004	−0.0003	−0.0003	−0.0001	−0.001	−0.001
	(0.0004)	(0.0004)	(0.0004)	(0.0005)	(0.0006)	(0.0006)
*FSA*	0.011 ***	0.012 ***	0.007 ***	0.009 ***	0.019 ***	0.020 ***
	(0.0017)	(0.0019)	(0.0019)	(0.0021)	(0.0029)	(0.0031)
*ELRA*	−0.036 ***	0.041 ***	0.042 **	0.072 ***	−0.064 ***	0.021
	(0.0141)	(0.0153)	(0.0209)	(0.0236)	(0.0189)	(0.0198)
ln*UR*	−0.189 ***	−0.140 ***	−0.197 ***	−0.082	−0.350 ***	−0.257 ***
	(0.0449)	(0.0489)	(0.0493)	(0.0556)	(0.0781)	(0.0820)
ln*PGDP*	0.494 ***	0.617 ***	0.449 ***	0.624 ***	0.414 ***	0.482 ***
	(0.0400)	(0.0435)	(0.0514)	(0.0580)	(0.0669)	(0.0702)
Constant	8.132 ***	1.209 ***	7.730 ***	0.649 *	9.748 ***	3.040 ***
	(0.2804)	(0.3053)	(0.3303)	(0.3725)	(0.5520)	(0.5798)
Fixed province and year	YES	YES	YES	YES	YES	YES
R-squared	0.3699	0.5211	0.4925	0.6638	0.4067	0.4872
F test	245.28 ***	129.64 ***	324.32 ***	76.27 ***	117.06 ***	72.88 ***
Hausman test	483.92 ***	50.27 ***	10.98 ***	137.61 ***	113.06 ***	21.45 ***
N	713	713	327	327	386	386

Table notes: * *p* < 0.10, ** *p* < 0.05, and *** *p* < 0.01, and the robust standard errors are indicated in parentheses.

**Table 2:** Baseline regression results of the SMGPC on NPY and CEY.

Variables	Overall		Positive Mismatch Region		Negative Mismatch Region	
	ln*NPY*	ln*CEY*	ln*NPY*	ln*CEY*	ln*NPY*	ln*CEY*
	(1)	(2)	(3)	(4)	(5)	(6)
*|SMGPC|*	0.017 *	0.014 *	−0.020 **	−0.035 ***	0.133 ***	0.149 ***
	(0.0095)	(0.0108)	(0.0095)	(0.0103)	(0.0197)	(0.0224)
*MCI*	−0.245 ***	−0.317 ***	0.046	−0.172 **	−0.329 ***	−0.389 ***
	(0.0317)	(0.0361)	(0.0664)	(0.0718)	(0.0378)	(0.0430)
*ADR*	0.006 ***	0.002 ***	0.002 ***	0.003 ***	0.001 **	0.001 **
	(0.0018)	(0.0004)	(0.0004)	(0.0005)	(0.0005)	(0.0006)
*FSA*	0.006 ***	0.007 ***	0.0038 **	0.006 ***	0.016 ***	0.017 ***
	(0.0018)	(0.0020)	(0.0019)	(0.0020)	(0.0028)	(0.0032)
*ELRA*	−0.083 ***	−0.005	0.026	0.056 **	−0.137 ***	−0.052 **
	(0.0142)	(0.0162)	(0.0211)	(0.0228)	(0.0181)	(0.0206)
ln*UR*	−0.443 ***	−0.394 ***	−0.468 ***	−0.353 ***	−0.543 ***	−0.451 ***
	(0.0453)	(0.0515)	(0.0495)	(0.0536)	(0.0751)	(0.0854)
ln*PGDP*	0.558 ***	0.681 ***	0.305 ***	0.4806 ***	0.515 ***	0.583 ***
	(0.0403)	(0.0459)	(0.0517)	(0.0607)	(0.0643)	(0.0731)
Constant	7.171 ***	0.247	8.306 ***	1.225 ***	8.402 ***	1.695 ***
	(0.2827)	(0.3216)	(0.3321)	(0.3594)	(0.5305)	(0.6035)
Fixed province and year	YES	YES	YES	YES	YES	YES
R-squared	0.4715	0.3667	0.4599	0.4043	0.5759	0.4723
F test	261.77 ***	125.67 ***	335.44 ***	97.66 ***	142.69 ***	69.35 ***
Hausman test	29.40 ***	23.43 ***	166.18 ***	15.83 **	36.64 ***	46.38 ***
N	713	713	327	327	386	386

Table notes: * *p* < 0.10, ** *p* < 0.05, and *** *p* < 0.01, and the robust standard errors are indicated in parentheses.

**Table 3:** The test results of the mediating effect of CLMS.

Sample Classification	Variables	ln*CLMS*	ln*NPA*	ln*CEA*	ln*NPY*	ln*CEY*
		(1)	(2)	(3)	(4)	(5)
Overall	*|SMGPC|*	0.071 ***	0.064 ***	0.061 ***	0.032 ***	0.029 ***
		(0.0104)	(0.0095)	(0.0104)	(0.0096)	(0.0110)
	ln*CLMS*		−0.213 ***	−0.215 ***	−0.212 ***	−0.213 ***
			(0.0341)	(0.0372)	(0.0344)	(0.0393)
	Control variables	YES	YES	YES	YES	YES
	Fixed province and year	YES	YES	YES	YES	YES
	R^2^	0.6980	0.4045	0.5436	0.4997	0.3931
	N	713	713	713	713	713
Positive mismatch region	*|SMGPC|*	0.086 ***	0.066 ***	0.063 **	−0.001	−0.003
		(0.0122)	(0.0098)	(0.0104)	(0.0099)	(0.0100)
	ln*CLMS*		−0.234 ***	−0.384 ***	−0.219 ***	−0.369 ***
			(0.0428)	(0.0455)	(0.0433)	(0.0440)
	Control variables	YES	YES	YES	YES	YES
	Fixed province and year	YES	YES	YES	YES	YES
	R^2^	0.7902	0.5384	0.7283	0.5022	0.5177
	N	327	327	327	327	327
Negative mismatch region	*|SMGPC|*	0.078 ***	0.133 ***	0.139 ***	0.149 ***	0.155 ***
		(0.0189)	(0.0201)	(0.0217)	(0.0198)	(0.0229)
	ln*CLMS*		−0.312 ***	−0.183 ***	−0.207 ***	−0.078 *
			(0.0550)	(0.0595)	(0.0541)	(0.0626)
	Control variables	YES	YES	YES	YES	YES
	Fixed province and year	YES	YES	YES	YES	YES
	R^2^	0.6378	0.4562	0.5005	0.5928	0.4746
	N	386	386	386	386	386

Table notes: * *p* < 0.10, ** *p* < 0.05, and *** *p* < 0.01, and the robust standard errors are indicated in parentheses.

**Table 4:** The test results of the mediating effect of CFAI.

Sample Classification	Variables	ln*CFAI*	ln*NPA*	ln*CEA*	ln*NPY*	ln*CEY*
		(1)	(2)	(3)	(4)	(5)
Overall	*|SMGPC|*	0.050 ***	0.007 **	0.007	0.016	0.017
		(0.0107)	(0.0027)	(0.0060)	(0.0063)	(0.0088)
	ln*CFAI*		0.841 ***	0.779 ***	0.668 ***	0.605 ***
			(0.0096)	(0.0213)	(0.0224)	(0.0310)
	Control variables	YES	YES	YES	YES	YES
	Fixed province and year	YES	YES	YES	YES	YES
	R^2^	0.3809	0.9490	0.8397	0.7723	0.5958
	N	713	713	713	713	713
Positive mismatch region	*|SMGPC|*	0.045 ***	0.011 ***	0.006	−0.048 ***	−0.065 ***
		(0.0118)	(0.0031)	(0.0052)	(0.0062)	(0.0069)
	ln*CFAI*		0.762 ***	0.799 ***	0.621 ***	0.658 ***
			(0.0147)	(0.0247)	(0.0298)	(0.0332)
	Control variables	YES	YES	YES	YES	YES
	Fixed province and year	YES	YES	YES	YES	YES
	R^2^	0.5289	0.9493	0.9253	0.7792	0.7418
	N	327	327	327	327	327
Negative mismatch region	*|SMGPC|*	0.125 ***	0.002	0.030 **	0.044 ***	0.076 ***
		(0.0224)	(0.0055)	(0.0139)	(0.0122)	(0.0191)
	ln*CFAI*		0.811 ***	0.752 ***	0.705 ***	0.576 ***
			(0.0126)	(0.0316)	(0.0278)	(0.0433)
	Control variables	YES	YES	YES	YES	YES
	Fixed province and year	YES	YES	YES	YES	YES
	R^2^	0.3743	0.9600	0.8030	0.8497	0.6480
	N	386	386	386	386	386

Table notes: ** *p* < 0.05, and *** *p* < 0.01, and the robust standard errors are indicated in parentheses.

## Data Availability

The original contributions presented in this study are included in the article/Supplementary Material. Further inquiries can be directed to the corresponding author.

## Supplementary Materials

The following supporting information can be downloaded at https://www.mdpi.com/article/10.3390/foods15101659/s1. Table S1: Inventory of elementary unit for agricultural non-point source pollution; Table S2: The carbon sources, emission coefficients, and reference sources for agricultural carbon emissions; Table S3: Chow test results for NPCE indicators (break year = 2016); Table S4: Endogeneity test results for using the instrumental variable method; Table S5: Robustness test results for excluding the atypical samples; Table S6: Robustness test results for shortening the panel data duration; Table S7: Robustness test results for changing the regression model; Table S8: Robustness test results for adopting an alternative grouping criterion; References [59,60,61,62,63,64,65,66,67] are cited in the Supplementary Materials.
