# Supplementary material for: Impact Assessment of Spatial Mismatch Between Grain Production and Consumption on Non-Point Source Pollution and Carbon Emissions from Grain Production in China
Source: Foods. 2026 May 9;15(10):1659. doi: 10.3390/foods15101659 (PMC13206509; doi:10.3390/foods15101659)
Supplement: Supplementary file 1 [file foods-15-01659-s001.zip › foods-4276268-supplementary.pdf]

## Supplementary Materials

### S1. Estimating grain consumption

Following the method for grain consumption estimation established in prior studies [59–61], this study disaggregated grain consumption into five categories: grain ration, grain feed, grain for industrial use, grain for seed use, and grain losses. Provincial grain consumption in China was estimated using the following formula:

$$C=C_r+C_f+C_i+C_s+C_l \quad \text{(S1)}$$

where $C$ denotes the total grain consumption; $C_r$ represents the grain ration consumption; $C_f$ refers to the grain feed consumption; $C_i$ indicates the grain consumption for industrial use; $C_s$ stands for the grain consumption for seed use; $C_l$ denotes the grain loss.

The grain ration consumption refers to the direct food consumption of urban and rural residents, including household grain consumption and out-of-home grain consumption. It was estimated as follows:

$$C_r=\frac{a_c}{1-r_{oc}}\times p_c+\frac{a_r}{1-r_{or}}\times p_r \quad \text{(S2)}$$

where $a_c$ and $a_r$ are the per capita household grain consumption of urban and rural residents, respectively; $r_{oc}$ and $r_{or}$ represent the proportion of out-of-home grain consumption for urban and rural residents, respectively; $p_c$ and $p_r$ denote the urban and rural population at the end of the year, respectively. Specifically, $r_{oc}$ and $r_{or}$ were adopted as 15% and 10%, respectively [60].

The grain feed consumption is the grain used to produce pork, beef, mutton, poultry meat, eggs, milk, and aquatic products for human consumption. It was estimated as follows:

$$C_f=\sum_{i=1}^{n} f_i\times\alpha_i \quad \text{(S3)}$$

where $f_i$ is the output of the $i$th food product, and $\alpha_i$ is the grain consumption conversion coefficient for the $i$th food product. The conversion coefficient for pork, beef, mutton, poultry meat, eggs, milk, and aquatic products was adopted as 2.01, 0.93, 0.81, 1.62, 1.72, 0.35, and 1.20, respectively [62].

The grain consumption for industrial use refers to the grain used as raw or auxiliary material for industrial use, including alcohol, beer, liquor, and other industrial products. The grain consumption for other industrial products was estimated as 25% of the total grain consumption for industrial use [60]. The grain consumption for industrial use was estimated as follows:

$$C_i=\sum_{i=1}^{n}\frac{d_i\times\gamma_i}{0.75} \quad \text{(S4)}$$

where $d_i$ is the output of the $i$th industrial product, and $\gamma_i$ is the grain consumption conversion coefficient for the $i$th industrial product. The conversion coefficient for alcohol, beer, and liquor was set at 3, 2.3 and 0.172, respectively [60].

The grain consumption for seed use was the grain reserved for reproduction. It was calculated as follows:

$$C_s=s_i\times\delta_i\div 1000 \quad \text{(S5)}$$

where $s_i$ and $\delta_i$ are the sown area and seed application rate per unit area of the $i$th grain crop, respectively. The seed application rate per unit area for rice, wheat, corn, and soybeans was

set at 41.7 kg/ha, 225 kg/ha, 225 kg/ha, and 78.9 kg/ha, respectively [60]. In addition, the grain consumption for tubers seed was accounted for at 10% of the total output of tubers.

Grain loss refers to the loss incurred during grain storage, transportation, processing, and other related processes, and was estimated at 5% of the total grain consumption [60].

**S2. Measurement of non-point source pollution from grain production**

Based on the classification framework of agricultural non-point source pollution, non-point source pollution from grain production is primarily attributed to fertilizer losses and agricultural solid waste emissions [6,63]. Accordingly, this study adopted the inventory analysis method to quantify the emissions of key pollutants, including chemical oxygen demand (COD), total nitrogen (TN), and total phosphorus (TP) generated from these two primary sources in grain cultivation [6,63]. Subsequently, these pollutants were converted into equivalent standard pollution loads in accordance with the Class II water standard specified in the National Environmental Quality Standard for Surface Water (GB 3838-2002) [37]. The specific inventory list is shown in Table S1. The corresponding calculation formulas were as follows:

$$NP=\lambda\sum_{j=1}^{n}NP_j=\lambda\sum_{j=1}^{n}\frac{P_j}{D_j} \qquad \text{(S6)}$$

$$P_{ij}=EU_{ij}\rho_{ij}(1-\eta_{ij})C_{ij}(EU_{ij},S) \qquad \text{(S7)}$$

where $NP$ is the equal-standard pollution load of non-point source pollution from grain production; $\lambda$ is the proportion of grain sown area to total crop sown area; $NP_j$ denotes the equal-standard pollution load of the $j$th pollutant; $P_j$ represents the total pollution discharge of the $j$th pollutant; $D_j$ refers to the $j$th pollutant's discharge concentration limit in water; $P_{ij}$ is the pollution discharge of the $j$th pollutant in unit $i$; $EU_{ij}$ signifies the indicator statistics of the $j$th pollutant in province $i$; $\rho_{ij}$ stands for the pollutant-producing coefficient in province $i$; $\eta_{ij}$ denotes the utilization efficiency of the related resources; $C_{ij}$ is the discharge coefficient of the $j$th pollutant in province $i$. The discharge concentration limits in water were set at 15 mg/L for COD, 0.5 mg/L for TN, and 0.1 mg/L for TP, as stipulated by the Class II water standard (GB3838-2002). The relevant resource utilization efficiency, pollutant-producing coefficients and pollutant-discharge coefficients were obtained from [64], and the First National Pollution Source Census.

**Table S1.** Inventory of elementary unit for agricultural non-point source pollution.

| Pollution source | Pollution units | Pollution indicator | Inventory of pollution |
| --- | --- | --- | --- |
| Fertilizer application | Nitrogen fertilizer, phosphate fertilizer, and compound fertilizer | Fertilizer consumption (t) | TN, TP |
| Agricultural solid waste | Rice, wheat, corn, beans, oil plants, and vegetables | Yield (t) | COD, TN, TP |

## S3. Calculation of carbon emissions from grain production

Following the emission factor method and measurement coefficients from existing studies [1,65,66], this study measured the carbon emissions from grain production by integrating six major direct and indirect carbon sources in the cultivation process, namely chemical fertilizers, pesticides, agricultural plastic films, agricultural diesel, irrigation, and tillage. Specifically, the provincial carbon emissions from grain production in China were calculated as follows:

$$CE=\lambda\sum_{i}^{n}E_i=\lambda\sum_{i}^{n}T_i\times\delta_i \qquad \text{(S8)}$$

where $CE$ denotes the total carbon emissions from grain production; $\lambda$ is the proportion of grain sown area to total crop sown area; $E_i$ refers to the carbon emissions from the *i*th carbon source; $T_i$ represents the amount of inputs from the *i*th carbon source; $\delta_i$ signifies the carbon emission coefficient of the *i*th carbon source. The list of carbon sources and corresponding carbon emission coefficients is presented in Table S2.

**Table S2.** The carbon sources, emission coefficients, and reference sources for agricultural carbon emissions.

| Carbon emission source | Carbon emission coefficient | Source |
|---|---|---|
| Chemical fertilizer | 0.8956 kgC/kg | Oak Ridge National Laboratory, USA |
| Pesticide | 4.9341 kgC/kg | Oak Ridge National Laboratory, USA |
| Agricultural film | 5.18 kgC/kg | Institute of Agricultural Resources and Ecological Environment, Nanjing Agricultural University |
| Diesel fuel | 0.5927 kgC/kg | 2013 IPCC United Nations Intergovernmental Panel on Climate Change |
| Irrigation | 20.476 kgC/hm$^2$ | [6,67] |
| Plowing | 312.60 kgC/hm$^2$ | School of Agronomy and Biotechnology, China Agricultural University |

## S4. Chow test results

**Table S3.** Chow test results for NPCE indicators (break year = 2016).

| Region | Statistic | NPA | NPY | CEA | CEY |
|---|---|---|---|---|---|
| National level | F | 204.87*** | 121.78*** | 246.40*** | 138.94*** |
| Eastern region | F | 137.39*** | 57.87*** | 105.20*** | 52.43*** |
| Central region | F | 118.14*** | 61.13*** | 146.88*** | 63.76*** |
| Western region | F | 403.71*** | 147.99*** | 263.16*** | 122.93*** |

Table notes: *$p$ <0.10, **$p$ <0.05, ***$p$ <0.01; degrees of freedom are F(2, 20) for each test.

## S5. Endogeneity and robustness test results

**Table S4.** Endogeneity test results for using the instrumental variable method.

| Sample classification | Variables | ln*NPA* (1) | ln*CEA* (2) | ln*NPY* (3) | ln*CEY* (4) |
|---|---|---|---|---|---|
| Overall | ∣*SMGPC*∣ | 0.056*** | 0.050*** | 0.018* | 0.012* |
| | | (0.0089) | (0.0100) | (0.0104) | (0.0128) |
| | Controls | YES | YES | YES | YES |
| | Province FE | YES | YES | YES | YES |
| | Year FE | YES | YES | YES | YES |
| | KP-LM statistic | 65.824*** | 65.824*** | 65.824*** | 65.824*** |
| | KP-LM Wald F statistic | 700.160*** | 700.160*** | 700.160*** | 700.160*** |
| | R-squared | 0.3694 | 0.5210 | 0.4715 | 0.3667 |
| | N | 713 | 713 | 713 | 713 |
| Positive mismatch region | ∣*SMGPC*∣ | 0.059*** | 0.040*** | -0.032*** | -0.050*** |
| | | (0.0101) | (0.0110) | (0.0102) | (0.0110) |
| | Controls | YES | YES | YES | YES |
| | Province FE | YES | YES | YES | YES |
| | KP-LM statistic | 36.738*** | 36.738*** | 36.738*** | 36.738*** |
| | KP-LM Wald F statistic | 305.041*** | 305.041*** | 305.041*** | 305.041*** |
| | Year FE | YES | YES | YES | YES |
| | R-squared | 0.4891 | 0.6628 | 0.4571 | 0.4001 |
| | N | 327 | 327 | 327 | 327 |
| Negative mismatch region | ∣*SMGPC*∣ | 0.120*** | 0.136*** | 0.153*** | 0.169 |
| | | (0.0239) | (0.0238) | (0.0224) | (0.0247) |
| | Controls | YES | YES | YES | YES |
| | Province FE | YES | YES | YES | YES |
| | Year FE | YES | YES | YES | YES |
| | KP-LM statistic | 74.216*** | 74.216*** | 74.216*** | 74.216*** |
| | KP-LM Wald F statistic | 610.012*** | 610.012*** | 610.012*** | 610.012*** |
| | R-squared | 0.4062 | 0.4868 | 0.5746 | 0.4710 |
| | N | 384 | 384 | 384 | 384 |

Table notes: **p* <0.10, ***p* <0.05, ****p* <0.01; robust standard errors are indicated in parentheses.

**Table S5.** Robustness test results for excluding the atypical samples.

| Sample classification | Variables | ln*NPA* (1) | ln*CEA* (2) | ln*NPY* (3) | ln*CEY* (4) |
|---|---|---|---|---|---|
| Overall | ∣*SMGPC*∣ | 0.047*** | 0.043*** | 0.011* | 0.006* |
| | | (0.0090) | (0.0100) | (0.0085) | (0.0099) |

| Sample classification | Variables | ln*NPA* | ln*CEA* | ln*NPY* | ln*CEY* |
|---|---|---|---|---|---|
| | | **(1)** | **(2)** | **(3)** | **(4)** |
| | Controls | YES | YES | YES | YES |
| | Province FE | YES | YES | YES | YES |
| | Year FE | YES | YES | YES | YES |
| | R-squared | 0.3816 | 0.5449 | 0.4973 | 0.3710 |
| | N | 621 | 621 | 621 | 621 |
| Positive mismatch region | \|*SMGPC*\| | 0.043*** | 0.028*** | -0.021** | -0.036*** |
| | | (0.0094) | (0.0107) | (0.0096) | (0.0104) |
| | Controls | YES | YES | YES | YES |
| | Province FE | YES | YES | YES | YES |
| | Year FE | YES | YES | YES | YES |
| | R-squared | 0.5075 | 0.6692 | 0.4737 | 0.4116 |
| | N | 315 | 315 | 315 | 315 |
| Negative mismatch region | \|*SMGPC*\| | 0.108*** | 0.117*** | 0.127*** | 0.135*** |
| | | (0.0192) | (0.0203) | (0.0165) | (0.0190) |
| | Controls | YES | YES | YES | YES |
| | Province FE | YES | YES | YES | YES |
| | Year FE | YES | YES | YES | YES |
| | R-squared | 0.4109 | 0.5137 | 0.6131 | 0.4989 |
| | N | 306 | 306 | 306 | 306 |

Table notes: *$p$ <0.10, **$p$ <0.05, ***$p$ <0.01; robust standard errors are indicated in parentheses.

**Table S6.** Robustness test results for shortening the panel data duration.

| Sample classification | Variables | ln*NPA* | ln*CEA* | ln*NPY* | ln*CEY* |
|---|---|---|---|---|---|
| | | **(1)** | **(2)** | **(3)** | **(4)** |
| Overall | \|*SMGPC*\| | 0.026** | 0.023* | 0.004* | 0.002* |
| | | (0.0116) | (0.0127) | (0.0125) | (0.0142) |
| | Controls | YES | YES | YES | YES |
| | Province FE | YES | YES | YES | YES |
| | Year FE | YES | YES | YES | YES |
| | R-squared | 0.3896 | 0.3683 | 0.5657 | 0.4265 |
| | N | 558 | 558 | 558 | 558 |
| Positive mismatch region | \|*SMGPC*\| | 0.007* | 0.004* | -0.044*** | -0.072*** |
| | | (0.0110) | (0.0133) | (0.0127) | (0.0142) |
| | Controls | YES | YES | YES | YES |
| | Province FE | YES | YES | YES | YES |
| | Year FE | YES | YES | YES | YES |
| | R-squared | 0.3847 | 0.4659 | 0.6044 | 0.5046 |
| | N | 244 | 244 | 244 | 244 |
| Negative mismatch region | \|*SMGPC*\| | 0.067*** | 0.080*** | 0.093*** | 0.107*** |
| | | (0.0241) | (0.0251) | (0.0248) | (0.0276) |

| | | | | | |
|---|---|---|---|---|---|
| | Controls | YES | YES | YES | YES |
| | Province FE | YES | YES | YES | YES |
| | Year FE | YES | YES | YES | YES |
| | R-squared | 0.4348 | 0.3929 | 0.5988 | 0.4880 |
| | N | 314 | 314 | 314 | 314 |

Table notes: *p <0.10, **p <0.05, ***p <0.01; robust standard errors are indicated in parentheses.

**Table S7.** Robustness test results for changing the regression model.

| Sample classification | Variables | ln*NPA* (1) | ln*CEA* (2) | ln*NPY* (3) | ln*CEY* (4) |
|---|---|---|---|---|---|
| Overall | \|*SMGPC*\| | 0.049*** | 0.044*** | 0.018* | 0.013* |
| | | (0.0093) | (0.0100) | (0.0094) | (0.0105) |
| | Controls | YES | YES | YES | YES |
| | N | 713 | 713 | 713 | 713 |
| Positive mismatch region | \|*SMGPC*\| | 0.044*** | 0.025** | -0.020** | -0.038*** |
| | | (0.0093) | (0.0105) | (0.0093) | (0.0100) |
| | Controls | YES | YES | YES | YES |
| | N | 327 | 327 | 327 | 327 |
| Negative mismatch region | \|*SMGPC*\| | 0.105*** | 0.122*** | 0.129*** | 0.144*** |
| | | (0.0201) | (0.0205) | (0.0193) | (0.0213) |
| | Controls | YES | YES | YES | YES |
| | N | 386 | 386 | 386 | 386 |

Table notes: *p <0.10, **p <0.05, ***p <0.01; robust standard errors are indicated in parentheses.

**Table S8.** Robustness test results for adopting an alternative grouping criterion.

| Sample classification | Variables | ln*NPA* (1) | ln*CEA* (2) | ln*NPY* (3) | ln*CEY* (4) |
|---|---|---|---|---|---|
| Overall | \|*SMGPC*\| | 0.043*** | 0.047*** | 0.013* | 0.017* |
| | | (0.0093) | (0.0101) | (0.0097) | (0.0109) |
| | Controls | YES | YES | YES | YES |
| | Province FE | YES | YES | YES | YES |
| | Year FE | YES | YES | YES | YES |
| | R-squared | 0.4047 | 0.5420 | 0.4632 | 0.3972 |
| | N | 616 | 616 | 616 | 616 |
| Positive mismatch region | \|*SMGPC*\| | 0.043*** | 0.031*** | -0.024** | -0.036*** |
| | | (0.0098) | (0.0109) | (0.0095) | (0.0103) |
| | Controls | YES | YES | YES | YES |
| | Province FE | YES | YES | YES | YES |
| | Year FE | YES | YES | YES | YES |
| | R-squared | 0.5112 | 0.6682 | 0.4561 | 0.4349 |
| | N | 286 | 286 | 286 | 286 |
| Negative mismatch region | \|*SMGPC*\| | 0.085*** | 0.118*** | 0.124*** | 0.157*** |
| | | (0.0213) | (0.0232) | (0.0216) | (0.0243) |

| | | | | |
|---|---|---|---|---|
| Controls | YES | YES | YES | YES |
| Province FE | YES | YES | YES | YES |
| Year FE | YES | YES | YES | YES |
| R-squared | 0.4284 | 0.5192 | 0.5595 | 0.4931 |
| N | 330 | 330 | 330 | 330 |

Table notes: **p* <0.10, ***p* <0.05, ****p* <0.01; robust standard errors are indicated in parentheses.
